# Effects of Team Emotional Authenticity on Virtual Team Performance

**DOI:** 10.3389/fpsyg.2016.01336

**Published:** 2016-08-31

**Authors:** Catherine E. Connelly, Ofir Turel

**Affiliations:** ^1^DeGroote School of Business, McMaster UniversityHamilton, ON, Canada; ^2^College of Economics and Management, South China Agricultural UniversityGuangzhou, China; ^3^Mihaylo College of Business and Economics, California State University FullertonFullerton, CA, USA; ^4^Department of Psychology, University of Southern CaliforniaLos Angeles, CA, USA

**Keywords:** virtual teams, teamwork behaviors, team trust, team emotional authenticity, online communication, distributed teams

## Abstract

Members of virtual teams lack many of the visual or auditory cues that are usually used as the basis for impressions about fellow team members. We focus on the effects of the impressions formed in this context, and use social exchange theory to understand how these impressions affect team performance. Our pilot study, using content analysis (*n* = 191 students), suggested that most individuals believe that they can assess others' emotional authenticity in online settings by focusing on the content and tone of the messages. Our quantitative study examined the effects of these assessments. Structural equation modeling (SEM) analysis (*n* = 81 student teams) suggested that team-level trust and teamwork behaviors mediate the relationship between team emotional authenticity and team performance, and illuminate the importance of team emotional authenticity for team processes and outcomes.

## Introduction

Virtual teams are common in organizations, because they allow a variety of members to participate despite geographic barriers (Hakonsson et al., 2016). Unfortunately, the very elements of virtual teams that make them convenient (i.e., communication takes place online instead of face-to-face) may also pose challenges for team performance (Hertel et al., 2005); that is, the extent to which team members have accomplished the goals or objectives set for them (Aube and Rousseau, 2011). For example, the use of lean media (e.g., technologies where the communication partners cannot see or hear each other, such as email, instant messaging, enterprise social networking tools) has been shown to increase unproductive conflict or conflict escalation (Friedman and Currall, 2003), information suppression between team members (Hedlund et al., 1998), and social loafing (Chidambaram and Tung, 2005). These challenges can make virtual teams difficult to manage and can diminish team performance (Turel and Zhang, 2010, 2011).

How can virtual teams be made more effective? Prior research has examined several elements of interpersonal interaction, and how they can be applied to explain virtual team dynamics and virtual team performance. For example, virtual teams have been shown to experience more task conflict (Massey et al., 2003) and less cooperation (Hakonsson et al., 2016) which can affect decision-making effectiveness (Hinds and Mortensen, 2005). However, the mechanisms through which misunderstandings among team members may occur and influence team performance remain unclear. One new variable which may explain team dynamics and performance is that of emotional authenticity, which is normally studied at a dyadic level and which captures service receivers' beliefs regarding the genuineness of the service provider (Grandey et al., 2007). Lower levels of perceived authenticity have been demonstrated to pose challenges to interpersonal interactions (Brotheridge and Grandey, 2002) and we believe this factor may also be important in team processes. We therefore introduce the concept of *team* emotional authenticity, which we define as the extent to which members of a team communicate with each other without suppressing or amplifying their emotional expression.

A second contribution of our study is the extension of emotional authenticity to the *virtual* team context. We build on research suggesting that people interacting online can and often do make assessments of the authenticity of their counterparts (Turel et al., 2013). People in such situations cannot see or hear the person with whom they are interacting when they form impressions of their communication partner's authenticity, which affects whether this person is perceived to be friendly (Turel et al., 2013). We believe that such assessments also have meaning in a team context, and we endeavor to understand the effects of these team-level aggregated impressions on team trust, teamwork behaviors, and team performance.

## Effects of team emotional authenticity in virtual teams

Virtual teams are typically comprised of several individuals who work on interdependent tasks, share responsibility for outcomes, and rely on technology for much of their communications (Webster and Staples, 2006). These teams are not new; a significant extant literature has demonstrated several reasons why they remain popular. First, they allow individuals with varying backgrounds (e.g., functional, geographical), to participate in decision-making processes, thereby providing new insights and improving team performance (Maznevski and Chudoba, 2000). Second, virtual team members also tend to participate more equally in discussions (Weisband, 1992). Indeed, several case studies show that the use of virtual teams can reduce costs, accelerate decision processes, and increase sales (e.g., May and Carter, 2001). Moreover, the increased prevalence of enterprise social networking tools allows for better collaboration between employees residing in different time zones (Jarrahi and Sawyer, 2013).

Despite the numerous logistical advantages of virtual teams, they are not universally successful. A significant body of research has investigated how virtuality may affect different forms of information sharing among team members, which may in turn affect team performance (Mesmer-Magnus et al., 2011). Other researchers have focused on characteristics of the team as well as the motivations of team members (e.g., Algesheimer et al., 2011; Gibson et al., 2011); the nature of the media (its “lean-ness”), employee adaptation and organizational form and incentives (Hakonsson et al., 2016). A key potential problems contributing to low performance of virtual teams is difficulties in interpersonal communication among team members. Online interactions tend to be shorter in length, have a somewhat quicker pace (Huang et al., 2008), and generate ambiguity regarding messages (Kock, 2007), which can lead to increased miscommunication. Silence (e.g., waiting for someone to respond), in particular, can be difficult for virtual team members to interpret, and can lead to interpersonal problems among team members that are difficult to resolve (Cramton, 2001).

In face-to-face exchanges, authenticity is commonly expected and assessed by message recipients (Ashforth and Tomiuk, 2000). It is typically evaluated through visual and auditory cues provided by the speaker. For example, subtle changes in facial expressions reliably differentiate authentic emotional displays from those that have been faked (Ekman et al., 1988). Similarly, facial characteristics can affect how someone's authenticity and trustworthiness is perceived (Baker et al., 2016). Vocal intonation and rhythm can also affect the extent to which verbal communications are judged to be honest (Banziger et al., 2009). Because these visual and auditory cues are not available in most popular communication technologies (e.g., text-messaging, email, social networking tools), users will need to rely on a different set of factors when trying to establish and evaluate the emotional authenticity of the colleagues with whom they are communicating. These text-based cues may be more difficult for virtual team-members to interpret accurately (Byron, 2008). Regardless, whether these cues are accurately or inaccurately interpreted, it is reasonable to assume that the formed assessment will influence employee behaviors, as humans expect and appreciate authentic communications (Grandey et al., 2005).

Consequently, members of virtual teams attempt to cope with the ambiguity imposed by physical and psychological distance by adapting their behaviors to the online context. For example, the content of the communication may take on greater importance; communicators may therefore provide more contextual information and err on the side of more detailed explanations. Online correspondents are reported to take particular care in their use of grammar and tone when communicating with people who they do not know well (Jessmer and Anderson, 2001; Vignovic and Thompson, 2010). Many people who communicate online use emoticons such as the 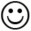 “smiley” or the;-) “wink,” to help clarify their meaning and add a more obvious emotional element to their messages. Indeed, there is evidence from fMRI brain activity scans that emoticons and non-verbal communication activate the same brain pathways (Yuasa et al., 2011).

### Authenticity

At the individual level, authenticity reflects the unreserved expression of one's true self in everyday life (Kernis and Goldman, 2006). To be “authentic” suggests that a person thinks, feels, and behaves in a manner that coincides with his or her most basic nature, and it is an important precursor to well-being (Schlegel et al., 2009). We can therefore distinguish inauthenticity from deception, which refers to “a message knowingly transmitted by a sender to foster a false belief or conclusion by the receiver,” and which can be accomplished through falsification, equivocation, or concealment (Buller and Burgoon, 1996, p. 205). Whereas, deception may involve both cognitive and affective elements (i.e., both facts and emotions) and is intentional, emotional authenticity refers specifically to emotions that are either amplified or suppressed and can be unintentional.

Emotional inauthenticity and deception have similar consequences; in interpersonal contexts, the perception that an individual is presenting him or herself in an authentic manner leads others to view this person as honest and sincere, which can promote trust, respect, and liking (Avolio and Gardner, 2005; Liu and Perrewe, 2006) and lead to a variety of desirable outcomes including service relationship satisfaction and commitment (Grandey et al., 2005; Hennig-Thurau et al., 2006). In contrast, people tend to react very negatively to inauthentic emotional displays such as fake smiles or insincere apologies (Grandey et al., 2005). As such, authenticity judgments are instrumental to the success of interpersonal interactions (Swann et al., 1994) and service relationships (Ashforth and Tomiuk, 2000). Similarly, depending on the perceived sincerity and appropriateness of the leader's emotional displays, followers may form either favorable or unfavorable impressions of the leader, which will, in turn, impact the extent of follower trust in the leader (Gardner et al., 2009). As organizations rely more on virtual teams, it is useful to consider how factors that have been important in other interpersonal interactions, such as emotional authenticity, might affect the performance of virtual teams which heavily rely on multi-party computer-mediated interactions.

As Grandey et al. (2007) note, authenticity is also separate from emotional labor, which refers to the efforts that individuals undertake in order to suppress or amplify the expression of their emotions (Brotheridge and Lee, 2003). Authenticity is possibly even more important in online contexts. The paucity of social cues in virtual communication means that each interaction can become disproportionately important. The perceived authenticity of these interactions becomes crucial, because virtual teams require positive interpersonal relationships in order to function effectively (Gully et al., 2002; De Dreu and Weingart, 2003).

We therefore introduce the concept of team emotional authenticity. It is conceptualized in our study as a team-level additive concept (Chan, 1998), which means that it captures the total level of the authenticity of emotional displays during the interactions between team members. Individual team members may be perceived to present different levels of authenticity, but the total level of their presumed authenticity is a team-level attribute (Kozlowski and Klein, 2000). For example, a team in which all members are perceived to be highly authentic will naturally have high levels of team emotional authenticity. In contrast, a team whose members who are all perceived to be slightly inauthentic will have a lower level of team-level emotional authenticity, and a team with a minority of members who are perceived to be very inauthentic will exhibit similarly low levels of team emotional authenticity.

It is not necessary for team members to agree on who among them is authentic or not; rather, it is the overall level of authenticity in the team that interests us. Our team-level conceptualization parallels those of other team-level constructs, such as teamwork behaviors (Tasa et al., 2007), team trust (e.g., Mach et al., 2010), and role overload (Marrone et al., 2007); in all of these cases there is no need to agree on who exactly behaved in a certain way and the meaning of the construct is through its description of a team attribute.

### Social exchange

Interactions within a team are often governed by social, rather than economic, exchanges. Team members rarely have the tools available to managers or supervisors to reward or influence others' behaviors (e.g., bonuses, promotion opportunities), so they must rely on reciprocal social exchanges. As noted by Blau (1964), social exchange theory describes a process in which any party who engages in positive behavior toward another will implicitly invoke a similar yet unspecified reciprocal behavior. The nature of these mutually rewarding exchanges expands over time, as trust is developed as obligations are met and new ones are created (Cropanzano and Mitchell, 2005). In a team environment, social pressures can heighten these feelings of obligation (Staples and Webster, 2007), as members seek acceptance, approval, or respect (Blau, 1964).

Social exchange has been previously applied to explain why positive treatment by team members is consistently related to increased team performance. For example, affective bonding between team members has been shown to predict the extent of their contributions to the team (Lin and Huang, 2010). Furthermore, the quality of relationships between team members has been linked to improved creativity (Liao et al., 2010), organizational citizenship behaviors (Love and Forret, 2008), and performance (Kamdar and Van Dyne, 2007). Although individual employee performance is primarily affected by these individuals' knowledge, skills, abilities, and motivation, team performance requires these attributes as well as the coordination of tasks among interdependent team members.

The role of social exchanges may be particularly important in self-managed virtual teams, because members' contributions are less likely to be closely monitored, and they have considerable discretion in terms of their behaviors (e.g., how much effort is expended; Chidambaram and Tung, 2005). In this context, factors that affect teams' perceptions and interpersonal dynamics will be a key determinant of team performance. As explained above, emotional authenticity is typically interpreted positively by communication partners (Avolio and Gardner, 2005; Grandey et al., 2005; Liu and Perrewe, 2006). Individuals prefer authentic communication, and expect it from others. As per social exchange theory, emotional authenticity would therefore elicit an obligation to reciprocate with further positive behaviors. Drawing on the social exchange and team performance literatures and focusing on the team level, we therefore expect a positive relationship between team emotional authenticity and team performance. This effect will likely be indirect, because team emotional authenticity is the trigger the drives assessments and reciprocation behaviors, which then translate into performance gains. We therefore hypothesize:

***Hypothesis 1*:** Team emotional authenticity will predict team performance.

The above-hypothesized effect may be mediated through social exchange processes and assessments. Trust is a key assessment in social exchange situations (Blau, 1964). Unlike economic exchanges, where the fulfillment of the obligation can be stipulated explicitly, social exchanges rely on parties trusting each other to reciprocate positive behaviors. We therefore expect that trust is an important component of the relation between team emotional authenticity and team performance.

More specifically, we argue that impressions of authenticity may be a basis (trust building cue) upon which trust perceptions within the team are formed. At the individual level, trust is a cognition regarding the trustworthiness of others, implying a willingness to be vulnerable to their actions (Mayer et al., 1995). At the team level, trust refers to a belief in the dependability and trustworthiness of team members (Zand, 1972; Dirks and Ferrin, 2001; Langfred, 2004).

Trust has been identified as being particularly important in virtual teams (Dennis et al., 2012). Nevertheless, trust does not arise in a vacuum. Trust is built, in part, based on past interactions and experiences as well as trust building cues (Zucker, 1986). As such, in virtual teams, trust is dependent on the behaviors of team members (Jarvenpaa et al., 2004) and the feedback that members receive from each other (Geister et al., 2006).

Extrapolating from research on emotional authenticity at the individual level (Grandey et al., 2005; Hennig-Thurau et al., 2006; Liu and Perrewe, 2006), we expect that teams who generally perceive their members to be authentic will associate this with higher levels of trustworthiness (e.g., honesty, integrity). In contrast, teams who generally perceive their members to be less authentic in their emotional displays (i.e., teams with members who are perceived to fake their emotions) will associate this with higher levels of dishonesty and deceitfulness and low levels of integrity. As noted above, these are important trust-building cues (Mayer et al., 1995). We therefore expect that teams whose members perceive each other to be less authentic in their emotional displays will doubt the trustworthiness (e.g., the integrity and benevolence) of its members and consequently develop lower team trust. Thus, team emotional authenticity is likely to positively predict team trust. We therefore suggest the following hypothesis:

***Hypothesis 2*:** Team emotional authenticity will increase team trust.

Trust has been widely established as a factor that can augment team performance (Jarvenpaa and Leidner, 1999; Piccoli and Ives, 2003; de Jong and Elfring, 2010). Its effect on performance is often mediated through process factors, such as organizational citizenship behaviors (Dirks and Ferrin, 2001). Trust reduces task and relationship conflict, and it improves team effectiveness (Curseu and Schruijer, 2010). Essentially, team trust enables the team to apply more effort toward the task instead of engaging in conflict (Jarvenpaa and Leidner, 1999; de Jong and Elfring, 2010), especially when individual autonomy in the team is low (Langfred, 2004). Trust among team members also increases the relational capital of the team and makes it easier for the team to overcome obstacles and challenging issues (Zornoza et al., 2009).

Although, much of the research involving trust and teams has been conducted with face-to-face teams, trust has also been shown to be critical to the success of virtual teams (Yakovleva et al., 2010). Interestingly, there is some evidence that trust is particularly difficult to achieve, yet crucial, when team members are not co-located (Staples and Webster, 2008; Yakovleva et al., 2010). Although, people are able to decipher many emotional cues from text-only communication (Cheshin and Rafaeli, 2009), it appears that trust can be affected by some aspects of the virtual team structure.

One possible outcome of reduced trust among team members is a commensurate reduction in teamwork behaviors, which are a collection of discretionary actions targeted at improving team performance (Tasa et al., 2007). This anticipated reduction is unfortunate, because teamwork behaviors have been repeatedly shown to be essential for increased team performance. These discretionary actions are performed by individuals within the group but are typically measured at the team level, and include interpersonal relationship management (e.g., conflict resolution) and self-management (e.g., goal-setting; Stevens and Campion, 1994). These behaviors capture an additive team attribute (e.g., some teams present more teamwork behaviors than others) that facilitates team collective efficacy and ultimately improved performance (Tasa et al., 2007). Teamwork behaviors are somewhat similar to person-focused organizational citizenship behaviors, which have been shown to be related to virtual team performance (Rico et al., 2011), but they encompass a broader set of behaviors and encapsulate a team attribute.

As noted by Dirks and Ferrin (2001), team trust influences the expectations regarding teammates' behaviors, so in high-trust teams motivation is directed toward team processes and goals rather than toward resolving issues which are peripheral to performance. Essentially, when individuals work in a high-trust team they are by definition more willing to be vulnerable to others, and will be more able to collaborate efficiently and effectively. Specifically, they will be more at ease performing teamwork behaviors, which can include risky behaviors such as discouraging off-topic conversations or calming down team members that are in conflict. The following hypotheses are therefore suggested:

***Hypothesis 3a*:** Team trust will predict teamwork behaviors.
***Hypothesis 3b*:** Team trust will mediate the relation between team emotional authenticity and teamwork behaviors.

As noted above, the interpersonal dynamics of a team may influence the extent to which its members are willing to contribute toward its goals, either by engaging in task performance or by ensuring that the team functions effectively. Indeed, teamwork behaviors have been identified as essential for high team performance (Stevens and Campion, 1994), because they contribute significantly to the team's completion of its tasks and goals (Tasa et al., 2007). For example, teams that ensure that all members participate, draw teammates into discussions, and resolve conflicts quickly, will outperform teams where a minority of members do most of the work, or engage in emotional conflict, or off-topic discussions.

Consequently, teamwork behaviors have clear relevance to virtual teams. Because of the increased potential for social loafing in teams that lack face-to-face contact, teamwork behaviors may be especially salient to team performance (Chidambaram and Tung, 2005). Teamwork behaviors are therefore proposed to mediate the relation between team-level trust and team performance. This is in line with prior research noting that team trust can indirectly influence team performance through team processes (Costa, 2003; Staples and Webster, 2008; Mach et al., 2010). We therefore suggest the following hypotheses:

***Hypothesis 4a*:** Teamwork behaviors will predict team performance.
***Hypothesis 4b*:** Teamwork behaviors will mediate the relation between team-level trust and team performance.

## Methods

The study was approved by the Institutional Review Board of California State University, Fullerton and the Ethics Review Board of McMaster University. All participants were over 18 years old and signed an approved consent form before participating in the study. Before testing our hypotheses, it was first important to establish if and how virtual teams form impressions of the emotional authenticity of their members. We therefore conducted a pilot study. A 6 week online collaboration exercise was conducted, involving sophomore students in an introductory course at a university in the United States. Two of the five sections were taught by one instructor and three were taught by another, but all sections covered the same course content as enforced by a course coordinator. The instructors randomly pre-assigned students to teams of three, consistent with team size norms in technology-supported teams research (Chidambaram and Tung, 2005). The students were asked to collaborate exclusively with an electronic collaboration tool to produce a report worth 5% of their final grade, which was required, as opposed to the completing of the surveys, which was optional and was encouraged with a small number of bonus points. An alternative bonus point opportunity (a short written assignment) was offered for students who did not want to participate in the study. The online tool mimicked existing enterprise collaboration solutions. It was a dedicated collaboration system thorough which individuals could securely and privately exchange text-based ideas and drafts, exchange document files, and develop their final submission. The system allowed participants to communicate asynchronously, and it did not enable them to exchange non-text files (e.g., videos) or use richer media (e.g., videoconferencing).

After the teams had been randomly assigned, each team member was asked to post a short message to his or her new team members. Specifically, participants were asked to state their name, describe their experience with team and on-line projects, their plans regarding the collaboration task and their enthusiasm regarding the project. Participants were told that details regarding the second part of the assignment would be provided after these initial introductions were completed.

After these initial messages were sent to the team members, each participant was asked to voluntarily quantitatively assess the emotional authenticity and trustworthiness of their virtual team members (time 1). Based on the work of Ekman et al. (1988), it was expected that even short encounters would be sufficient for team members to make an assessment of their team members' emotional authenticity. Descriptive statistics and demographic variables were also captured at this time (age, gender, Internet experience, frequency of use of online communications, and years of work experience). The completion of the survey was voluntary and was encouraged with bonus grade points. For the qualitative analysis, participants were asked to describe how they can tell from someone's electronic communications (e.g., emails) if they are being genuinely friendly or if they are only acting that way (i.e., “faking” their friendliness).

### Pilot study and bases for emotional authenticity assessments in virtual teams

The total enrollment across the five course sections was 197 students, who were randomly assigned to 65 teams (Three students per team, with two exceptions of four-member teams). Response rates in the first and second data collection rounds were high (97 and 93%, respectively), alleviating the concern of non-response bias. To reduce the potential biasing effects of prior familiarity on individual assessments, only individuals who stated that they did not know their teammates prior to the assignment (checked via the survey) were retained for further analysis. The sample size in the first wave of data collection was consequently reduced from 191 to 171. However, all 191 responses were used for the qualitative analysis. They contained 190 usable responses.

The textual responses were subjected to content analysis following the Krippendorff (1980) procedure. This procedure requires that an a-priori codebook be created that can be used to categorize textual communication, so that raters can then determine the frequency with which examples of each category appear. We applied this procedure to the within-team textual communication of the participants in the study. First, a codebook was developed and refined by one of the researchers, based on a review of the responses (see Appendix in Supplementary Material). It was then used independently by two external raters to classify the responses into categories. The raters were asked to classify responses based on a detailed code schema (third level) if possible. In cases where this was impossible, they were instructed to use a less detailed coding schema (second level) that aggregates codes at the third level. Inter-rater agreement was then analyzed at both the third and second code levels.

The initial classification yielded raw agreement rates of 78 and 83% at the third and second code levels, respectively. The initial Cohen's Kappas (agreement adjusted for agreement due to chance) were 0.77 and 0.78 (both significant at *p* < 0.001) at the third and second code levels, correspondingly. These are acceptable starting points for inter-rater agreement levels. The raters then met and discussed their differences. Agreement was achieved at the most detailed level for all items but ten. As a result, the post-discussion Cohen's Kappas were 0.91 and 0.96 at the third and second code levels, correspondingly. The frequencies in the Appendix in Supplementary Material include only items for which agreement was obtained. It therefore reflects a reliable categorization of participant opinions regarding sources they use for authenticity assessments in online settings.

According to our respondents, there are two primary factors that contribute to communication being assessed as emotionally authentic: presentation style (48%), and content (17%). Within the “content” category, the dominant subcategory was the disclosure of personal information (8%), including “additional information about self, above and beyond what is necessary (Respondent #38).” The presentation style category can be further subdivided into two categories: message format (30%), which included word choice (16%) and tone (15%), as well as the use of emoticons (3%) and punctuation (3%); and writing style (18%). Even though communications were asynchronous, response speed (2%) did not appear to be a dominant determinant of emotional authenticity.

### Main study

Continuing the task that was described in the pilot study, teams were asked to collaborate using an electronic communication tool over a period of 3 weeks to produce a short report about the privacy issues raised by Apple's iPhone. The task related to content covered in class and relied on students' ability to analyze research and integrate their personal opinions. The assignment had no single or obvious solution; the task was judgmental in nature (Bonner et al., 2002). After completing this task in week 6, individuals were invited to voluntarily complete a second survey. Team performance was assessed independently in the seventh week by the course instructors (not affiliated with this research project and blind to it) who evaluated the assignments; one final grade was assigned for each team.

#### Sample

Surveys collected in both phases (after the introductions and after the task completion) were matched using student identifiers. In order to capture team-level phenomena, only records belonging to teams with at least two responses across the two study phases were retained. As a result, the final dataset included 241 individuals, who belonged to 81 teams (three teams of two members, 77 teams of three members, and one four-member team, representing 85% response rate at the team level. The final sample was comprised of almost equal proportions of men and women (118 and 123, respectively). Respondents' ages ranged from 18 to 44 (mean age of ~23) and they had, on average, 10 years of Internet experience. Students were quite familiar with online communication tools (e.g., instant messaging, social networking websites, email, blogs) and used such tools several times a week to several times a day.

#### Measures

All measures were adapted from previously validated instruments, and used seven-point Likert-type scales anchored with “strongly disagree” (1) and “strongly agree” (7).

Team-level authenticity (time 1, during week 2) was measured by asking participants to rate their teammates' authenticity, in order to avoid self-report bias. These ratings were collected anonymously, but then aggregated for each team member, and then compiled for each team. Because it has been suggested that inauthentic emotions can involve suppression of felt emotions as well as their artificial amplification (Glomb and Tews, 2004), we added two reverse-coded items to the two items suggested by Grandey et al. (2005). The added items are “I believe this teammate is withholding his or her true emotions and feelings regarding this project” and “I believe this teammate is expressing emotions (e.g., enthusiasm regarding the project) in his or her message he or she does not truly feel.” The internal consistency of this four-item scale was high (α = 0.82 from teammate 1; α = 0.85 from teammate 2).

Interpersonal trust (time 1, during week 2) among teammates was measured with a four item scale adapted from McAllister et al. (2000). A sample item of this measure is “based on the introduction message, he or she can be trusted.” The internal consistency of this scale was high (α = 0.95 from teammate 1; α = 0.96 from teammate 2).

Teamwork behaviors (time 2, during week 6) were assessed using a scale developed by Tasa et al. (2007). A representative item is “[He/She] participated in developing strategies to achieve team goals.” The internal consistency of this scale was high (α = 0.95 from teammate 1; α = 0.97 from teammate 2). Because teamwork behaviors are measured by team members' reflections on their observations of their team members, they are captured at time 2.

Team performance (time 3, week 7) was measured with each team's grade for their team's assignment. The assignments were assessed with consistent criteria: the extent to which privacy issues were identified, the integration of technical and legal insights, and the clarity of the exposition and argumentation. The task related to content covered in class and relied on students' ability to analyze research and integrate their findings and personal opinions. To account for the fact that report submissions were graded by one of two instructors, a standardized *z*-score was calculated for all grades, separately for each instructor. This procedure adjusts grades by taking into account deviations from the central grading tendency (i.e., easiness or toughness) of each instructor, and ensures that the dependent variable captures relative performance rather than the idiosyncrasies of each instructor. The instructors were blind to the objectives of the study.

Several control variables were also included in the surveys. First, because gender diversity can influence team outcomes (Mohammed and Angell, 2004), we calculated the Blau's index for each team in the sample (Harrison and Klein, 2007). This index ranges from 0 to 1 (total homogeneity to total heterogeneity). Note that while surface diversity is obvious in face-to-face settings, it may not be that noticeable in online settings where names are the primary method by which a team member's gender may be determined. Because some names are uncommon or unisex, the effect of this diversity may be reduced in online settings. Nevertheless, we accounted for it.

Second, because interaction intensity can be indicative of commitment, engagement, motivation, and effort, it may influence team performance over and above the hypothesized effects. We therefore controlled for team interaction intensity, which was captured at week 6 in the second survey. This construct encapsulates the extent to which individuals were engaged in online interaction, and it was manifested by (1) the self-reported frequency with which individuals checked their online collaboration space, and (2) the self-reported frequency with which individuals posted content on their online collaboration space. Both items used a seven-point Likert-type scale anchored with “never” (1) and “very often” (7). These measures were averaged to the team level. The internal consistency of this scale was adequate (α = 0.74).

Third, within-team heterogeneity in interaction intensity may be indicative of social loafing or merely unbalanced effort (i.e., when some members reported high frequency of checking and posting messages, and others did not). Such unbalanced efforts can also influence team members' motivation (downward spiral), and performance (Latané et al., 1979). We therefore controlled for within-team heterogeneity in interaction intensity, which was operationalized as the within-team standard deviation in interaction intensity scores (Harrison and Klein, 2007).

## Analysis and results

The research model is at the team level of analysis. Team performance, within-team heterogeneity in interaction intensity, and gender diversity were captured at the team level, but our other variables required the aggregation of individual-level data (i.e., peer assessments and self-reports) to the team level. To do this, index scores were first created for each construct for each team member. Because constructs have demonstrated sufficient reliability at the individual-level with Cronbach's alpha values exceeding 0.70, we used the average of all items as a proxy for the latent concept that the construct captures.

Because we are examining the additive team constructs (Chan, 1998) of authenticity, trust, and teamwork behaviors, our aggregated variables do not require a certain threshold of Intra-class correlations (ICC) values (LeBreton and Senter, 2008). The measures used for these concepts refer to individual team members, but the meaning of these constructs is derived from their average or overall level within the team. We do not demonstrate agreement among team-members, because disagreement does not infringe the validity of the construct (e.g., see treatment of team-level teamwork behaviors in Tasa et al., 2007). Essentially, it is the total amount of the construct (e.g., teamwork behaviors, authenticity) that is the focus of interest; high levels in one individual may counterweigh the low levels contributed by another individual without compromising the validity of the variable. As such, these constructs were operationalized by taking the by-team mean of the index scores of the relevant constructs. The means, standard deviations, and correlations are provided in Table 1. The diagonal includes individual level construct reliabilities as pertaining to team member 1 (top) and team member 2 (bottom), which were assigned randomly.

**Table 1:** **Descriptive statistics, internal consistencies, and correlations**.

	**Mean**	**SD**	**(1)**	**(2)**	**(3)**	**(4)**	**(5)**	**(6)**
1. Team-level authenticity (time 1)	5.57	0.63	(0.82)					
			(0.85)					
2. Team-level trust (time 1)	5.42	0.79	0.49^**^	(0.95)				
				(0.96)				
3. Team-level teamwork behaviors (time 2)	4.46	0.94	0.40^**^	0.54^**^	(0.95)			
					(0.97)			
4. Team performance (time 3)	0.00	0.99	0.05	0.02	0.28^*^			
5. Gender diversity (time 1)	0.34	0.19	0.03	0.01	0.14	−0.09		
6. Interaction intensity (time 2)	3.68	0.54	0.12	0.12	0.22	0.23	−0.13	(0.74)
7. Heterogeneity in interaction intensity (time 2)	0.52	0.29	−0.21	−0.28^*^	−0.26	−0.02	0.19	−0.27

* *p < 0.05*,

** *p < 0.01*.

Even though ICC scores are not needed for construct validation (see paragraph above), we calculated them for the team-level variables for descriptive purposes. The ICC scores for authenticity [ICC(1) = 0.029, *p* = 0.37], trust [ICC(1) = 0.139, *p* = 0.055], and teamwork behaviors [ICC(1) = 0.65, *p* < 0.001] indicated that different raters perceived each individual team member as having different levels of authenticity and slightly different trustworthiness. In contrast, there is strong agreement regarding the teamwork behaviors presented by individuals, possibly because team members adjusted their efforts to these of other teammates (Latané et al., 1979), or merely because teamwork behaviors were easier to observe and decipher. The within-person ICC was also calculated, to see whether each team member consistently gives low (or high) authenticity, trustworthiness, and teamwork behavior ratings to others. These results [authenticity: ICC(K) = 0.60, *p* < 0.001; trust: ICC(K) = 0.59, *p* < 0.001; teamwork behaviors: ICC(K) = 0.181, *p* < 0.01] suggest that some individuals have a tendency to rate others' authenticity and trustworthiness a certain way, and that their ratings of their team members are affected by this tendency.

**Figure 1:**
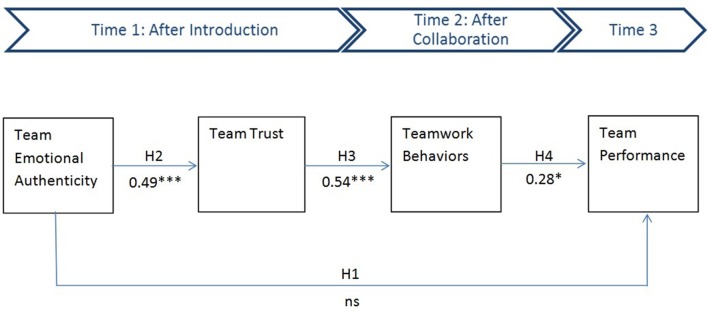
**Structural model**.

After these preliminary checks, a model with all hypothesized relationships as well as the three control variables (gender diversity, interaction intensity, and within-team heterogeneity in interaction intensity) was specified and estimated using AMOS 23.0. The results of this model are shown in Figure 1. Structural Equation Modeling-based path analysis indicated that this model fits the data very well [$\chi_{\left(8\right)}^{2}$ = 9.71 (non-significant, *p* < 0.29), CFI = 0.96, IFI = 0.97, RMSEA = 0.052 with *p*-close = 0.43, and SRMR = 0.051]. While all three mediation paths pertaining to the research model were significant at least at *p* < 0.01, the direct effect of team emotional authenticity to team performance was not significant. Hence, although hypothesis 1, suggesting that team emotional authenticity will predict team performance, was not supported, hypotheses 2, 3a, and 4a were supported. Team emotional authenticity predicted team trust, team trust predicted teamwork behaviors, and teamwork behaviors predicted team performance. In addition, the effects of the control variables were not statistically significant, with *p*-values ranging from 0.12 to 0.34. For reasons of parsimony, the control variable effects were removed. Consequently, the more parsimonious model was specified and estimated. This revised model also fit the data well [$\chi_{\left(2\right)}^{2}$ = 4.02 (non-significant, *p* < 0.14) CFI = 0.95, IFI = 0.95, RMSEA = 0.08 with *p*-close = 0.19, and SRMR = 0.059].

Next, we removed the non-significant direct effect path between team emotional authenticity and team performance, and re-estimated the model. The model presented good fit [$\chi_{\left(3\right)}^{2}$ = 4.32 (non-significant, *p* < 0.23) CFI = 0.97, IFI = 0.97, RMSEA = 0.07 with *p*-close = 0.31, and SRMR = 0.057] and all paths were significant (at least at *p* < 0.01). Hence, the results are consistent across the models. A chi-square difference tests suggest that there are no significant changes in the chi-square values between the models (*p* < 0.56). Hence, only for parsimony reasons, the model without the direct path from team emotional authenticity and team performance is deemed better.

Because a significant initial relationship between independent and dependent variables is not necessary to establish mediation, we continued with our analyses to determine if mediation was present (Preacher and Hayes, 2008). Mediation was tested using bootstrapping with 500 re-samples in AMOS 23 (Cheung and Lau, 2008). For the hypothesis where team trust mediates the relation between team emotional authenticity and teamwork behaviors, the standardized indirect effect was 0.19; 95% CI [0.06; 0.34], *p* < 0.006. For the hypothesis where teamwork behaviors mediate the relation between team trust and team performance, the standardized indirect effect was 0.15; 95% CI [0.04; 0.35], *p* < 0.02. Given these *p*-values, support was therefore found for hypotheses 3b and 4b. Team-level trust mediated the relation between team emotional authenticity and teamwork behaviors. Teamwork behaviors mediated the relation between team-level trust and team performance.

The results imply that trust and teamwork behaviors fully mediate the effect of team emotional authenticity on team performance. This mediational process was validated with the abovementioned bootstrapping procedure. The standardized indirect effect of team emotional authenticity on team performance was 0.06 and was significant at *p* < 0.02 with 95% CI [0.01; 0.18]. At the same time, the standardized direct effect of team emotional authenticity on team performance was not significant (*p* < 0.56). This supports the emerged full mediation process.

We also tested two *post-hoc* models in which (a) trust is removed and (b) teamwork behaviors are removed and trust predicts performance directly. In the first model, the team authenticity-teamwork behavior (β = 0.37) and teamwork behavior-performance (β = 0.29) paths were again significant (*p* < 0.01). This implies that perhaps trust is not always needed for translating team authenticity assessments into performance gains; authenticity by itself can promote teamwork behaviors. In the second model, the team authenticity-trust (β = 0.41) was significant (*p* < 0.001) but the trust-performance effect was not (*p* < 0.24), which implies that trust mediates only the translation of team authenticity into teamwork behaviors, but not the direct translation of team authenticity into actual performance gains. Team performance appears to necessarily require teamwork behaviors.

## Discussion

This paper investigated how perceptions of emotional authenticity affect the performance of virtual teams. Our qualitative pilot study enabled us to ascertain that team members can make assessments of the emotional authenticity of their electronic communication counterparts (accurate or not), and it provided some insights into how these assessments are made. Furthermore, the results of the quantitative study suggest that perceptions of emotional authenticity have significant effects on virtual team performance, albeit indirectly. Our research is the first to conceptualize the construct of team-level emotional authenticity, and examine its effects on the performance of virtual teams. By introducing a new team-level variable that may influence the effective functioning of a team, we are able to explain variance in team performance; virtual teams, and possibly other types of teams, may be able to perform well if the members are able to interact with each other in a way that demonstrates emotional authenticity.

### Theoretical contributions

Because of the inherent challenges in using virtual teams effectively, a growing body of research has emerged that seeks to identify factors that affect these teams' performance (Hertel et al., 2005). This literature has predominately focused on how certain factors (e.g., team characteristics, nature of the tasks, organizational context, supervisory behaviors) affect team outcomes, as mediated by factors such as team interaction and team behaviors (Martins et al., 2004; Powell et al., 2004; Webster and Staples, 2006). We extend this line of work by focusing on a new yet possibly important team-level variable, emotional authenticity. We hence contribute to a growing body of research that suggests that team characteristics and initial behaviors can influence team processes and, ultimately, team performance (Jarvenpaa and Leidner, 1999; Maznevski and Chudoba, 2000; Montoya-Weiss et al., 2001), and point to overlooked characteristics which have the potential to influence team performance.

The results of our first study suggest that most people can and possibly often do form impressions of a communication partner's emotional authenticity, even in online settings where they do not have access to the visual and auditory cues taken for granted in face-to-face communication (e.g., tone of voice, facial expressions). Indeed, < 15% of the respondents stated that it is impossible to assess authenticity without these cues. This reaffirms findings advanced in past research (Turel et al., 2013), but our research focuses on teams and not dyads. It therefore implies that a closer attention should be given to the information and outcomes of emotional authenticity assessments in teams, and especially virtual or distributed teams.

According to our findings, virtual team members base their emotional authenticity assessments on several factors (e.g., content, writing style). However, it is interesting to note that participants did not base their impressions entirely on the presence or absence of a single factor, such as an “emoticon.” Rather, they appeared to synthesize the impact of several factors along a continuum (e.g., too much enthusiasm was suspect, but too little was also not viewed positively). Interestingly, participants also formed their impressions based on the congruence between the presence and absence of different cues throughout a particular message or across several messages. Participants made higher-order assessments of the emotional authenticity of the communication partner, by comparing their experiences with how they expected their team members to behave; i.e., fulfill their socially learnt “role” of team members as per role theory (Solomon et al., 1985).

### Limitations and directions for future research

Because the true intentions of each communicator are unknown, it is possible that the team members' impressions are partially, or even completely unfounded. In other words, team members may be inferring inauthenticity when a team member was actually sincere, or they may believe that their team members are authentic when their expressed emotions were, in fact, incongruent with the emotions that they were experiencing. However, the possibility that these impressions may be unfounded does not preclude these perceptions from still affecting the way in which team members interact with each other. As with perceptions of justice or trustworthiness, perceptions of emotional authenticity are “in the eye of the beholder,” and are meaningful for the person who holds them. Because people have the capacity to form impressions of the emotional authenticity of their virtual team members, we are able to proceed with our second study, where we empirically examine the consequences of these impressions.

Our research suggests that people form impressions of their teammates' emotional authenticity even when they cannot see or hear the other person. These impressions are driven by the content and style of the message. Future research can use experiments to determine which of these factors are most important in creating the impression of inauthentic emotional expression, and how the influence of these factors is mitigated or exacerbated by the context (e.g., the urgency or the sensitivity of the communication) and by characteristics of the communicator or the recipient (e.g., personality, self-efficacy, familiarity with the technology).

Much of the existing research about the effects of authentic emotional expression has been conducted in the context of customer service encounters (e.g., Grandey et al., 2005) or other interpersonal relationships involving dyads (e.g., Swann et al., 1994). Our research is the first to extend the study of authenticity to the team context. However, additional research would be useful to further indicate the specific types of teams in which emotional authenticity affects team performance. For example, in less interdependent teams, or in teams with a clear leader, interpersonal communication may be less crucial for encouraging team trust or teamwork behaviors. It would also be interesting to examine how asymmetries in team-level authenticity may affect the effective functioning of the team. Although we examined team's authenticity from an additive perspective (i.e., the total level of the authenticity perceived by all team members), it is possible that teams with a high consensus about the authenticity of the emotions expressed by the team (a direct consensus construct per Chan, 1998) would have higher levels of team trust or performance than teams where some team members believe that other members are authentic but others believe that they are being insincere or fake. It would similarly be interesting to examine the consequences of significant gaps between self-assessments and peer-assessments of authenticity. We examined peer assessments in our study, because we were interested in their effects on team trust, teamwork behaviors, and team performance. However, differences between someone's self-reported authenticity and how they are perceived by others may also have serious consequences for interpersonal relationships and should be examined in future research. Likewise, it would be interesting to examine how assessments of authenticity evolve over time; communication partners may reassess their judgments as they collect new information from repeated interactions, or they may be biased by strong “first impressions.” Future research can explore this issue further.

An important body of research is also emerging regarding predictors of team trust. For example, some authors have shown that one's propensity to trust is a significant factor in predicting how much team members trust each other (Yakovleva et al., 2010). Other research has examined how different aspects of an email message, such as grammatical errors or etiquette deviations, can affect how trustworthy the writer appears (Vignovic and Thompson, 2010). Trust research also examines content-based antecedents such as justice (Dirks and Ferrin, 2001). The dominant view in the literature is that trust is based on perceptions of the other party's (or parties') ability, benevolence, and integrity (Mayer et al., 1995). Our findings pinpoint an important predictor of such trustworthiness beliefs; people should ensure that they communicate in a way that is perceived to be emotionally authentic. Thus, our findings indicate a new antecedent of team-level trust. These findings also set the stage for further research that identifies additional consequences of authentic emotional expression in a team context. For example, it would be interesting to see if less authentic displays affect team members' perceptions of interpersonal justice, or if they result in higher levels of socio-emotional conflict between members, or lower levels of team satisfaction. Future research can extend these findings, to determine how both parties' individual differences interact with the characteristics of the messages to affect the assessments that are made of the writer's emotional authenticity.

Our research also underscores the importance of teamwork behaviors as a crucial antecedent of team performance. Although, trust was important in our model, teamwork behaviors mediated the relationship between trust and performance. Perhaps because of the extensive collaboration and coordination inherent in interdependent teams, the prosocial behaviors were necessary for effective performance. Future research can explore in more detail how authenticity may predict other prosocial behaviors in other contexts (e.g., organizational citizenship behaviors).

One potential limitation of this research is the fact that our participants were students. Although, students are not uncommon participants in team research studies (e.g., Colquitt et al., 2002; Tasa et al., 2007), it is important to acknowledge that these respondents are younger than many virtual team members who work in organizations, and they are also very familiar and comfortable with the technologies being used in our study. However, we should also note that our sample is relevant to the relationships that we investigate; our participants were asked to complete tasks (e.g., evaluate the authenticity of a team member; complete an assignment) that were within the scope of their abilities, and that addressed our research questions. Similarly, the independent measure of performance was highly relevant to this population (assignment grade). Although future research may replicate our results in a field setting, our findings provide a useful basis for future investigations.

A further limitation of our study stems from the fact that assessments of authenticity are necessarily subjective. It is likely that different individuals will gauge a particular individual's authenticity differently. Nonetheless, we should emphasize that it is the effects of authenticity perceptions, rather than authenticity itself, that is most interesting to us. When a group perceives a certain level of authenticity, this appears to affect its behaviors, whether or not these perceptions are well-founded.

Despite the limitations of our sample, it should be noted that our study has several positive features; we were able to capture an external measure of team performance, the task was meaningful to the research participants and was identical for all teams, data were collected at three points in time, and the authenticity of each team was assessed by the other members of his or her team (i.e., we did not rely on self-reports).

It is also important to note that none of the team members in our study knew each other before they participated in our experiment, and they did not interact with each other face-to-face while the experiment was ongoing (a 6 week period). These conditions were important for us to establish so that we could ensure that the authenticity impressions were based on the online interactions rather than on any prior or in-person communications. However, many virtual teams are comprised of subsets of members who are co-located, and many virtual teams meet periodically in a face-to-face setting (Webster and Staples, 2006). It has further been argued that technology-mediated communications can complement interactions that take place face-to-face (Dixon and Panteli, 2010). Now that the effects of virtual-only communication have been established, it will therefore be interesting to replicate and extend our findings with virtual teams that have some members who know each other well, or who see each other in person from time to time.

### Practical implications

Considering that online communication tools offer considerable cost savings and other practical advantages, our results are promising, in that they suggest that online communication has the potential to be perceived as emotionally authentic. This research therefore points to the importance of ensuring that employees are provided with training about how to communicate in ways that are both convincing and authentic in their online encounters, in virtual teams, or merely in their daily routines. Individuals tend to overestimate their ability to communicate emotions effectively via email (Kruger et al., 2005). As such, organizational policies regarding technology-mediated communication should be examined and revised, if necessary. For example, many corporate policies prohibit certain forms of technology-mediated communication (e.g., the use of ALL CAPS, the use of emoticons), or provide “canned” answers to be used by service personnel (Turel et al., 2013). However, our results suggest that there may be advantages to giving employees some degree of latitude when communicating via lean media such as email in order to allow them to pass as more emotionally authentic. Moreover, workshops for teaching employees about the importance of authenticity in online communications and ways to improve such assessments by other may be developed. Previous research has found that management practices that have traditionally been applied to managing individuals (e.g., goal setting) have the potential to be useful when applied to the virtual team context (Hertel et al., 2004) and our study adds to this body of evidence.

## Conclusion

This research examines a novel factor in the success of virtual teams: the extent to which team members are perceived to express their emotions authentically, or “team emotional authenticity.” Our empirical findings describe some of the factors that affect perceptions of emotional authenticity, and suggest that it affects team trust, which in turn affects teamwork behaviors, which then improves team performance. As we continue to see further innovations in electronic collaboration tools, such as enterprise social networking tools and messaging services on smartphones, virtual teams are likely to become even more popular. Research on how emotional authenticity is communicated in this context and an understanding of how it affects the success of virtual teams, will only become more important.

## Author contributions

CC and OT participated equally in study conceptualization, execution, analysis, and write-up.

## Funding

The authors gratefully acknowledge the research funding for this project provided by The Social Sciences and Humanities Research Council of Canada (SSHRC).

### Conflict of interest statement

The authors declare that the research was conducted in the absence of any commercial or financial relationships that could be construed as a potential conflict of interest.
