# Supplementary material for: Effects of Team Emotional Authenticity on Virtual Team Performance
Source: Front Psychol. 2016 Aug 31;7:1336. doi: 10.3389/fpsyg.2016.01336 (PMC5005960; doi:10.3389/fpsyg.2016.01336)
Supplement: Supplementary file 1 [file DataSheet1.docx]

**Appendix: Code Book and Frequencies for Online Emotional Authenticity Assessments ***

| **Main Category** | **Sub Category (Second Level)** | **Detailed Category (Third Level)** | **Definition/Examples** |
| --- | --- | --- | --- |
| 1. Content (16.7%) | 1.1 Partner Related (12.2%) | 1.1.1 Contact information (1.1%) | Any mention of additional contact information provided, such as email or phone number, or encouraging additional contact such as “contact me anytime”. |
|  |  | 1.1.2 Personal Information (8.3%) | Mention of partner revealing past experiences, or additional information about self, above and beyond what is necessary; refers to general information or general responses referring to “personal experiences”, “Personal life” or “opening themselves up”, “past experiences” or “revealing personality” in their messages; references to mention of personal life, school, work, likes or dislikes. |
|  |  | 1.1.3 Emotions (2.2%) | Mention of partner discussing emotions they (the partner) are experiencing in general or towards project or interaction, such as excitement or enthusiasm. |
|  | 1.2 Project Related (1.1%) | 1.2.1 Future Planning (1.1%) | Any reference to partner planning for future communication or other project-related behaviors; offering to help in the future; future planning can relate to project or interactions in general. |
|  | 1.3 Reader Related (3.3%) | 1.3.1 Personal comments (0.6%) | Any mention of personal comments aimed at the reader, such as “good luck” or “nice to meet/work with you”. |
|  |  | 1.3.2 Introduction/Closing (2.8%) | Any mention of originality or content of introductory or closing salutations; standard/formal salutations are associated with a lack of genuineness; more original or personalized salutations are associated with perceptions of genuineness. |
| 2. Response speed (2.2%) | 2.1 Response speed (2.2%) | 2.1.1 Response speed (2.2%) | Any reference to length of time it took partner to respond (shorter response time positively associated with perceived genuineness). |
| 3. Presentation Style  (48.3%) | 3.1 Message Format (30%) | 3.1.1 Word choice (16.1%) | Any reference to choice of words, diction or vocabulary used by partner; includes responses such as “wording of the communication” , “words they use”, “words like excited” or “word usage”, or “how they use their words”. |
|  |  | 3.1.2 Tone (15%) | Any reference to tone of partner’s correspondence. |
|  |  | 3.1.3 Punctuation (3.3%) | Any reference to use of punctuation, such as exclamation points or all capitals. |
|  |  | 3.1.4 Use of Symbols (2.8%) | Any reference to use of symbols or emoticons, such as happy faces. |
|  |  | 3.1.5 Length of message (2.8%) | Any reference to the length of message, but not concerning detail level, clarity or consistency. |
|  |  | 3.1.6 Format (0.6%) | Any reference to the format of the message, such as use of bullet points (negative) or full paragraphs (positive); sentence structure. |
|  |  | 3.1.7 Presentation (1.1%) | Any reference to spelling, grammar, or overall quality of the presentation of message. |
|  | 3.2 Writing Style (18.3%) | 3.2.1 Clarity (1.7%) | Refers to flow of message, how easy or natural it appears or is to read; responses such as “easy to read”, “natural”, or “flows well”; may also refer to clarity, or ease of understanding of what the partner is trying to communicate. |
|  |  | 3.2.2 Consistency of message (5%) | Refers to consistency of message, either over a number of communications or within same message across paragraphs, or even tone or language; considering post or information in post within context; can also refer to consistency between written information and behavior. |
|  |  | 3.2.3 Exaggerated language (2.8%) | Negative code; Reference to use of exaggerated language, symbols, punctuations, overemphasis on certain terms, points or over reliance on symbols, punctuation to convey friendliness and/or excitement; negatively related to perceived genuineness of partner. |
|  |  | 3.2.4 Detail (2.8%) | Refers to level of detail of message or correspondence in general, but not length; can refer to attention to detail, or to partner explaining or describing material rather than just stating it, either general or project-related |
|  |  | 3.2.5 Directness (0.6%) | Refers to the partner’s tendency to not skirt around an issue but get right to the point, be honest and open about status of project; not being passive aggressive; not writing more than is necessary. |
|  |  | 3.2.6 Joking (0.6%) | Reference to partner’s use of jokes or humor. |
|  |  | 3.2.7 Clichés (0.6%) | Reference to partner’s use of clichés. |
|  |  | 3.2.8 Appears honest (0.6%) | Reference to partner appearing to be open and honest or sincere. |
| 4. Not applicable (16.7%) | 4.1 Not possible (14.4%) | 4.1.1 Not possible (14.4%) | Reader does not believe it is possible to gauge genuineness via online communication only. |
|  | 4.2 General Belief (0.6%) | 4.2.1 General Belief (0.6%) | Reader’s general belief in sincerity of people; reader’s tendency to assume people are sincere, genuine and/or honest. |
|  | 4.3 Longer time needed (1.1%) | 4.3.1 Longer time needed (1.1%) | Reader believes more time or repeated exposure to partner’s electronic communication is needed to make a determination regarding partners’ genuineness. |
|  | 4.4 Not enough information (0.6%) | 4.4.1 Not enough information (0.6%) | Not enough information provided in answer to determine whether or not reader believes it is possible to gauge genuineness via online communication; response lacks sufficient clarity or context to be decipherable. |

** The percentage of level-two codes can be more than the sum of level-three codes because raters coded at level two in cases where the preferred level-three code was not obvious or could not be agreed upon. All categories do not add to 100% due to rounding.*
